# The dark side of private medical education in Brazil

**DOI:** 10.3389/fmed.2025.1504794

**Published:** 2025-02-17

**Authors:** Bruno B. Andrade

**Affiliations:** ^1^The BRIGHT Lab, Multinational Organization Network Sponsoring Translational and Epidemiological Research (MONSTER) Institute, Salvador, Brazil; ^2^Laboratory of Clinical and Translational Research, Gonçalo Moniz Institute, Oswaldo Cruz Foundation, Salvador, Brazil

**Keywords:** medical education, Brazil, medical training, private medical centers, academic performance

## Introduction

The rapid expansion of private medical education in Brazil, often touted as a solution to healthcare access issues (1), has exposed a darker reality that demands urgent attention (2). As someone actively involved in academic administration and research within several private medical schools in this country, I have witnessed first-hand the consequences of this unregulated growth. What was supposed to be a step forward in addressing the country's shortage of healthcare professionals has instead given rise to a medical education crisis that is threatening the very fabric of our healthcare system.

**Figure 1:**
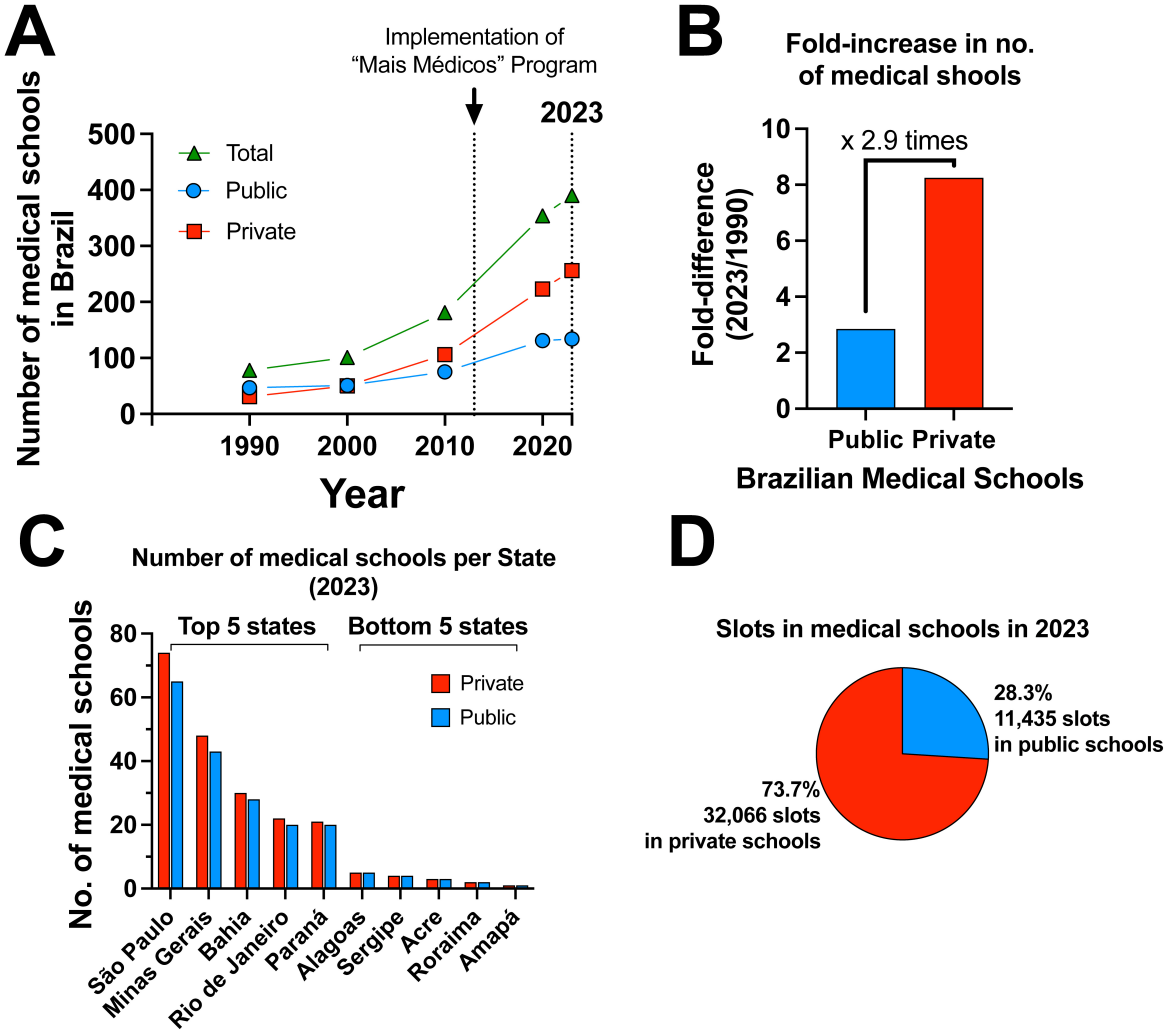
Growth and distribution of medical schools in Brazil. **(A)** The number of medical schools in Brazil from 1990 to 2023; **(B)** Fold-increase in the number of medical schools from 1990 to 2023; **(C)** Distribution of available slots in medical schools in 2023; **(D)** Number of medical schools by state in 2023. Data source in (3).

Over the past decade, the number of medical schools in Brazil has grown significantly, with private institutions driving much of this expansion (1). Figure 1 highlights this trend, showing a sharp rise in the total number of medical schools from 1990 to 2023, largely due to the burgeoning private sector (Figure 1A). During this period, growth in private medical schools nearly tripled that of public institutions (Figure 1B). This rapid expansion, particularly accelerated by the “Mais Médicos” program, reflects the increasing role of private institutions in shaping medical education in Brazil. However, regional disparities remain stark: Southeastern states like São Paulo and Minas Gerais host the highest concentrations of medical schools, while Northern states such as Amapá and Roraima have far fewer institutions (Figure 1C) (3). By 2023, private medical schools accounted for 73.7% of all available slots, offering over 32,000 positions, compared to just 28.3% provided by public institutions. This shift underscores the dominant role of private institutions in meeting the growing demand for medical education in Brazil.

**Figure 2:**
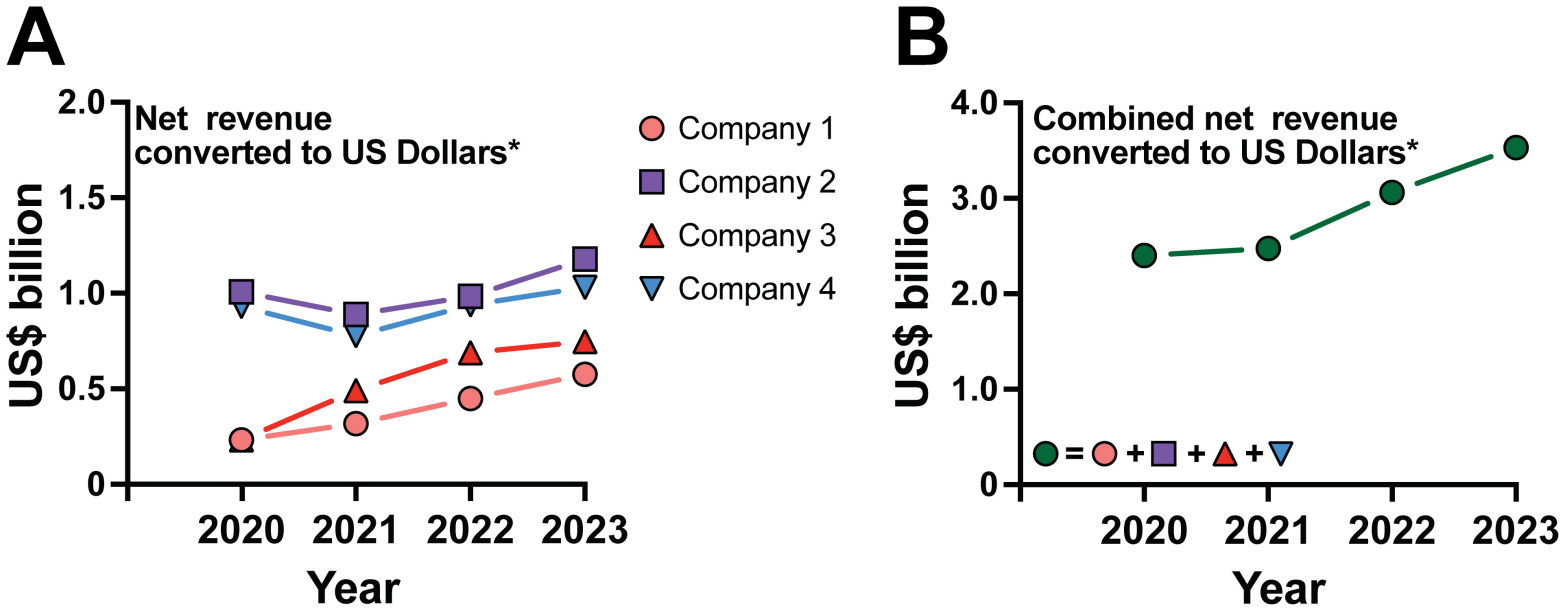
Net revenue growth of major private education conglomerates in Brazil (2020–2023). **(A)** Individual revenue growth for four companies from 2020 to 2023; **(B)** Combined net revenue of all four companies over the same period. Net revenue is displayed in billions of US dollars for the largest private education conglomerates in Brazil. Data source in (4–7). The US Dollar values were calculated by dividing the revenues in Reais by the average annual exchange rate for each respective year (data source for Annual average exchange rates: IPEADATA: http://www.ipeadata.gov.br/Default.aspx). This method ensures a more accurate reflection of the companies' financial performance adjusted to the historical economic context.

Private institutions in Brazil are expanding at an unprecedented rate, driven by significant increases in revenue. Figure 2 highlights this rapid growth, presenting the net revenue (in billions of US dollars) of four of the largest private education conglomerates in Brazil from 2020 to 2023. The data, sourced from the official websites of these conglomerates (4–7), reveal a sharp upward trajectory in revenue generation. This consistent growth is evident both in the individual performance of each company (Figure 2A) and in their combined net revenue (Figure 2B). These trends underscore the increasing profitability and aggressive expansion of private medical education providers. While the growth in net revenues supports the claim of rapid proliferation and market dominance, it is essential to interpret this data with caution. The reported revenues encompass a broad range of operations, including general higher education and digital solutions, in addition to medical education. Despite this diversity, analyzing revenue trends over this 4-year period offers valuable insight into the collective market size and financial trajectory of these companies, highlighting the substantial scale of their operations in the medical education sector.

This rapid expansion of private medical institutions raises serious concerns about the quality of education offered, as there is often little regard for the academic preparedness of incoming students or the quality of education being provided (2, 8–11). The opening of medical schools in small towns, where the healthcare system is unprepared and unable to support students or provide the necessary academic experience, often lacks sufficient qualified professors or instructors to teach, mentor, and supervise the training of future doctors (1). Local infrastructure plays a crucial role in medical education, as the training of future physicians relies on appropriate clinical settings for students to complete their education. However, according to the Federal Council of Medicine, 78% of the municipalities hosting medical schools in Brazil lack the necessary infrastructure for proper professional training (12). These areas face challenges such as insufficient hospital beds, a shortage of medical teams, inadequate Family Health services, and a lack of teaching hospitals (12). Furthermore, 73% of municipalities applying to receive new medical schools are similarly unprepared to adequately train future healthcare professionals (13). In this scenario, the result is a system where the primary focus is on enrolling as many students as possible, with insufficient attention paid to proper academic and clinical training (14). The core mission of training competent doctors is frequently being overshadowed by financial incentives, leaving us to face the consequences of a poorly trained medical workforce (15).

## A system motivated by profit

Brazil's demand for healthcare professionals, combined with gaps in regulatory oversight and favorable governmental policies, has encouraged private investors and education conglomerates to seize the opportunity of turning medical education into a highly money-spinning business model (14). This shift has resulted in a market-driven system in which private institutions charge exorbitant tuition fees, often exceeding US $32,000.00 annually [approximately seven times the country's minimum wage; (16)], limiting access to medical education to those who can afford it. To accommodate the rising costs, private loans and government-sponsored programs such as the Brazilian Student Financing Fund for Higher Education (FIES) and the University for All Program (PROUNI) have emerged as alternatives to improve accessibility (17). However, these mechanisms have contributed to high levels of debt for graduates, which, in my opinion, influence their career decisions (18). In my experience, many graduates, burdened by this debt, are forced to prioritize immediate employment in high-demand, high-stress environments such as emergency care or to pursue less rigorous postgraduate programs that offer flexible schedules but do not provide the same level of clinical training as residency (15). A more comprehensive, nationwide analysis is needed to validate these perceptions and guide the development of effective policies.

More importantly, this issue extends beyond access. Although a few excellent private medical schools exist across the country's diverse economic macro-regions, many institutions still lack the necessary infrastructure and academic rigor to provide high-quality medical education (1, 14). Numerous private medical schools operate under substandard facilities, often lacking proper teaching hospitals or outpatient settings (14), both of which are critical for hands-on clinical training. In such cases, practical training is often arranged through business agreements with external healthcare providers (15). Albeit one might expect that such poorly equipped schools would face difficulties operating, regulatory adjustments have been made to meet the urgent need for more physicians in underserved regions (14). One such adjustment is the Education-Health Public Action Organizational Contract [COAPES; (19)], established by the Ministries of Health and Education. COAPES is a key component of the “Mais Médicos” program and was introduced as a framework for strengthening partnerships between medical schools and local healthcare services to improve the quality of medical training and healthcare delivery. However, its implementation has been inconsistent, with many institutions struggling to meet the intended goals due to limited resources or weak partnerships with healthcare services. These and other adjustments, while addressing immediate physician shortages, have also allowed for the establishment of institutions with minimal quality standards, which in turn complicates efforts to recruit experienced professors and ensure that students receive comprehensive medical training.

## An oversupply of underprepared graduates

One of the most glaring issues with the current system is the oversupply of medical graduates who are inadequately prepared for the workforce (20). On the surface, it seems that the demand for residency positions far surpasses the supply, leaving many graduates without the advanced training necessary to practice medicine independently. However, a deeper analysis shows that only half of the available residency positions, especially in family medicine in remote areas, are filled (20). This suggests that many Brazilian medical graduates are deliberately choosing not to pursue residency training (21).

Facing financial pressure, many new medical graduates in Brazil opt for immediate employment in high-risk areas such as emergency or critical care, where residency is not required (22). However, these roles expose them to extreme working conditions for which they are often unprepared, posing risks to both their wellbeing and patient safety. A recent study from our group carried out with Emergency Medicine residents in Brazil demonstrated that inexperience and the complexity of cases exacerbate uncertainty in clinical decision-making, particularly during the first year of residency (23). First-year residents reported significantly higher levels of stress and difficulty handling uncertainty compared to their more experienced peers, which impacts both professional judgment and patient outcomes (23). This issue becomes even more critical in rural and/or underserved regions, where healthcare resources are already scarce. In these areas, these undertrained doctors, including many of these new graduates, often become the only available providers (8, 14). Importantly, this not only fails to alleviate the healthcare shortage but worsens it by flooding the market with inadequately trained professionals (15). As a result, both patients and new doctors are left vulnerable to the consequences of insufficient training and support.

Moreover, while medical residency is a crucial component of comprehensive medical training, fewer doctors are opting for it likely due to the high cost of medical education, years of unpaid study, and the demanding structure of residency in Brazil (21). Residents receive a stipend equivalent to just three shifts worked by a non-specialist yet endure exhausting workloads and many years of training (24). Despite an increase in the number of undergraduate medical spots, the growth in residency positions, especially in high-paying areas, has not kept pace. This imbalance has created a parallel market where students, already in debt from medical school, pay for preparatory courses for residency exams (25). Even when successful, they face years of low pay, fueling the rise of profit-driven *Lato Sensu* postgraduate programs [for definitions of such programs, please see Box 1; (26)]. These programs, which allow doctors to tailor their work and study schedules, often charge exorbitant fees for training that lacks sufficient practical experience (25). While they provide access to the specialized care market, they also devalue the profession, leaving many specialties undercompensated (26). Recently, the Brazilian Federal Council of Medicine declared that such *lato sensu* programs do not confer the title of specialization (27). There is an urgent need for stricter regulation of these programs to ensure proper training and protect the integrity of medical practice in the country. For better context, Box 1 illustrates the differences in medical postgraduate training pathways in Brazil, comparing medical residency, *stricto sensu* and *lato sensu* postgraduate programs (28, 29).

**Box 1:** Differences in postgraduate medical training pathways in Brazil.

**Medical residency**	***Stricto Sensu* postgraduate programs**	***Lato Sensu* postgraduate programs**
• A form of postgraduate characterized by in-service training. It operates under the responsibility of health institutions, either university-affiliated or not, and is supervised by highly qualified medical professionals with strong ethical and professional standards. • A regulated practical training program lasting 2 to 5 years, depending on the specialty. Upon completion, the physician receives the title of specialist recognized by the Federal Medical Council.	• Master's and doctoral programs focused on academic research, typically lasting 2 to 4 years. While these courses provide a solid theoretical foundation, they do not automatically grant specialist certification.	• Short-term specialization programs (minimum of 360 h), generally aimed at professional development in specific areas. However, they do not confer a recognized specialist title, according to the Federal Medical Council.

## The regional imbalance

Another issue exacerbating this problem is the geographic concentration of medical schools and healthcare professionals in Brazil's wealthier regions (21). Private institutions tend to cluster in the more affluent Southeast, leaving the North and Northeast regions, where healthcare shortages are most acute, with minimal coverage (1). As a result, the regions most in need of well-trained medical professionals remain underserved, perpetuating a cycle of inequity and inadequate access to healthcare in these areas (1). Additionally, the chronic shortage of qualified professors in the interior further compromises medical education in underserved areas (30). To address this, increasing internship opportunities in rural regions is crucial to ensure students receive hands-on experience and encourage graduates to consider careers in these underserved areas, aligning medical training with the healthcare needs of Brazil.

Moreover, graduates from private institutions are less likely to practice in underserved regions due to the allure of higher-paying opportunities in urban centers (1). This misalignment between where doctors are trained and where they are needed the most reflects a broader systemic failure.

## The erosion of academic excellence

The quality of medical education cannot be measured solely by the number of graduates produced. It is deeply connected to academic rigor, clinical training, and a commitment to research and innovation (30, 31). However, private medical schools often deprioritize research, a critical component in cultivating a well-rounded and knowledgeable healthcare workforce (11). This disparity may contribute to findings that public medical schools in Brazil consistently outperform private institutions in national evaluations, underscoring the urgent need for standardized quality assurance measures across all medical education providers (14).

From my experience in academic administration and mentoring students, I have seen the transformational power of integrating research into medical education. Students who engage in research are better equipped to understand complex medical issues, also contributing to advancements in the field (32, 33). However, those private institutions focused mostly on profit frequently neglect research opportunities, limiting the intellectual and professional growth of their students (34–37). A recent study highlighted that although the majority of medical students feel motivated to engage in research, only a minority actively participate in research activities, with inadequate guidance and time constraints referred as significant barriers (33). In this context, structured mentoring programs have proven to be an effective solution, as evidenced by our group in a study on scientific mentoring in medical schools (38). These programs not only increase student satisfaction and engagement, but also improve research outcomes, with 27.5% of advised students publishing their final papers in academic journals or conferences (38). Therefore, in our experience, integrating scientific mentoring and promoting involvement in research from an early stage of the course is crucial to promoting comprehensive medical education and future academic success, and can be considered a successful strategy.

This reduction in research focus also impacts Brazil's ability to address major public health challenges. The country faces significant healthcare burdens, including chronic diseases like diabetes (39) and infectious diseases such as dengue (39). Another notable example is tuberculosis, a disease that imposes a significant financial burden on Brazil (39). A recent study by our group demonstrated that Brazil, which remains classified as a high-burden country for tuberculosis, is unlikely to achieve the targets outlined in the World Health Organization's End TB Strategy (40). To combat these issues, we need medical professionals who are trained clinicians and researchers capable of advancing our understanding and treatment of these conditions.

## A call for reform

The challenges presented by the unregulated growth of private medical schools are systemic. To address this crisis, Brazil must implement stronger regulatory frameworks to ensure that all medical graduates meet minimum, but good, standards before entering the workforce. One potential solution is the introduction of a nationwide examination for medical graduates, similar to those already used for the legal profession in the country. Such an exam could serve as a critical quality control measure, ensuring that only those who are sufficiently trained are allowed to practice medicine. Recently, Brazilian congressmen and the Federal Council of Medicine have decided to move forward with implementation of this initiative, which could represent a significant step toward improving the quality and consistency of medical education in the country (41).

Addressing the unequal distribution of medical professionals must extend beyond simply increasing the number of graduates each year. It requires a focus on the complex, systemic factors, such as inadequate work infrastructure, security and poor access to essential services, that deter healthcare professionals from relocating to underserved areas, even as competition for work in the countries' wealthiest centers intensifies (1). To truly resolve this issue, policies must aim to create more attractive working and living conditions in underserved regions, ensuring that healthcare professionals have the support and resources they need to thrive both personally and professionally.

Most importantly, private institutions must be held to higher standards. Schools that fail to meet academic and clinical requirements should face consequences, including the revocation of their licenses. Incentives should be introduced to encourage these institutions to pursue external accreditation, ensuring they meet globally recognized benchmarks. If we are to preserve the integrity of the medical profession in Brazil, the focus must shift to producing high-quality professionals, rather than simply increasing the number of graduates. The Brazilian government also has to re-evaluate the current standards to grant approval for establishment of such schools. Ultimately, it is the state's responsibility to ensure that the population has access to well-trained doctors, by upholding stringent standards for medical education and prioritizing quality over quantity in the approval of new institutions.

## Contextualizing Brazil's medical education crisis: regional and global perspectives

The challenges facing Brazil's private medical education system mirror broader trends observed across the Global South, with important regional and global implications. Comparisons with other countries in Latin America, as well as BRICS nations, highlight systemic issues tied to rapid educational expansion, economic disparities, and workforce maldistribution. These parallels provide valuable insights into potential strategies for reform and underline the global relevance of Brazil's crisis.

Across Latin America, the privatization of higher education has followed a similar trajectory, often prioritizing profit over quality (42, 43). Countries like Ecuador, Peru and Colombia have experienced rapid increases in medical school enrollment, driven by private sector growth. However, much like Brazil, these nations grapple with insufficient oversight and uneven educational quality. In Ecuador, the significant rise of private universities offering medical degree programs has posed challenges in maintaining educational quality and adequately preparing professionals. Postgraduate medical students in Ecuador also face scenarios of insufficient training and uneven distribution of healthcare workers, mirroring challenges seen in Brazil (44). In Peru, studies highlight the proliferation of for-profit medical schools with inadequate infrastructure, resulting in underprepared graduates and significant geographic disparities in healthcare access (44). Similarly, Colombia has faced challenges balancing the increasing demand for healthcare professionals with the need to maintain high standards in medical education (45).

Despite these struggles, some regional initiatives offer lessons for Brazil. For instance, Chile's stringent accreditation process for medical schools ensures consistent quality and alignment with national healthcare priorities. These measures have been effective in reducing disparities in healthcare provision, particularly in underserved areas (46).

An additional issue requiring attention is the migration of Brazilian medical students to study abroad. According to the Ministry of Foreign Affairs, over one-third of Brazilian medical students pursue their education in Latin America, with Argentina, Bolivia, and Paraguay among the most popular destinations (47). This trend is driven by the pursuit of affordable medical education, often coupled with more flexible admission requirements. Paraguay, in particular, has emerged as a key destination due to its lower tuition fees. However, this growing migratory flow raises significant concerns about the quality of medical education provided in these countries. A striking example is the difficulty many Brazilians who graduated abroad face when attempting to revalidate their degrees through the “Revalida” Exam, which recognizes foreign medical qualifications. Alarmingly, data reveal that 85.6% of Brazilian graduates from Paraguayan institutions fail this exam (48), highlighting a substantial gap in educational standards and regulatory oversight. These discrepancies pose a serious risk to the competence and readiness of future medical professionals trained abroad.

Similar challenges are evident in other nations, such as India and South Africa within the BRICS grouping, offering instructive case studies on managing medical education quality. India, for example, has also witnessed an explosion of private medical colleges, many of which prioritize revenue over educational rigor. Reports indicate that these institutions frequently lack essential teaching hospitals and clinical training opportunities, echoing challenges seen in Brazil (49). Nevertheless, India's introduction of a nationwide exit exam for medical graduates, the National Exit Test (NExT), aims to standardize competency assessments and improve graduate preparedness (50, 51). As mentioned above, Brazilian governmental authorities are discussing the establishment of a similar exam to address the inconsistencies in medical training quality (41).

South Africa, on the other hand, has focused on integrating community-based education into medical curricula, ensuring that students gain practical experience in underserved areas (52). This approach enhances the relevance of medical training and encourages graduates to work in rural regions post-qualification (53, 54). Adopting community-oriented training frameworks could help Brazil address its geographic maldistribution of healthcare professionals.

The broader Global South offers further insights into addressing inequities in medical education. Countries like India, Iran, Phillippines, Malaysia and Thailand have successfully implemented public-private partnerships to expand medical training capacity while maintaining quality standards (55–61). These models emphasize collaboration between government bodies and private institutions to align educational outcomes with national healthcare needs.

Moreover, many nations in the Global South prioritize affordability and accessibility. In Cuba, for instance, medical education remains state-funded and universally accessible, ensuring a steady supply of well-trained healthcare professionals for domestic and international service. While Brazil's context differs significantly, adopting elements of Cuba's approach, such as government subsidies tied to service commitments in underserved areas, could help balance the dual goals of equity and quality (62–64).

Situating Brazil's medical education crisis within these broader contexts underscores the need for targeted reforms. Regional and global comparisons reveal potential strategies, including stringent accreditation processes, standardized competency exams, community-based education, and innovative public-private partnerships. Learning from these examples can help Brazil not only address its internal challenges but also position itself as a leader in medical education reform within the Global South.

## Final remarks

Brazil is facing a profound crisis in its medical education system. The unchecked expansion of private medical schools (21), driven by financial gain rather than a commitment to academic excellence, has created a warning sign for the future of our healthcare system. The rise of large healthcare networks and outsourcing in the public health system is contributing to an unstable employment market, where short-term contracts and hiring doctors as private contractors are becoming the norm (14). These trends erode job security, prevent long-term professional development, and create a disconnect between healthcare providers and the public health mission of the Brazilian Unified Health System [Sistema Único de Saúde, SUS; (14)]. If we continue on this path, we risk producing a generation of underqualified doctors as well as undermining the very foundation of our healthcare system.

To move forward, we must shift our focus back to quality. This means adopting more stringent criteria for selecting students who will enter medical schools, investing in proper infrastructure, prioritizing research, ensuring academic rigor, and implementing regulatory reforms that protect the integrity of medical education. Only by addressing these issues can we hope to build a healthcare system that truly serves the needs of all Brazilians.
